# Vaccines don't save lives, vaccination does

**DOI:** 10.1016/j.lanwpc.2021.100099

**Published:** 2021-01-27

On Dec 14, 2020, Singapore became the first Asian country to approve a COVID-19 vaccine. The availability of COVID-19 vaccines raises hope for curbing the pandemic and resuming some kind of social and economic normalcy. The success of vaccine-based protection strategies largely relies on sufficient vaccine coverage within a population, and alongside the ongoing debate on equitable vaccine access, the question of how many people are willing to receive the vaccine has been raised.

The answer to this question looks broadly promising for the Western Pacific region. According to a vaccine confidence survey by the World Economic Forum and Ipsos in December, 2020, 80% of Chinese participants, 75% of Australian and South Korean participants, and 60% of Japanese participants were willing to receive a COVID-19 vaccine if it became available. Similar positive rates were reported in two Australian vaccination intention surveys done in April and June, 2020, which were published in *The Lancet Infectious Diseases*, and a survey of 13 426 people from 19 countries in a *Nature Medicine* study published on Oct 20, 2020. However, the data from a few countries do not represent the full picture of COVID-19 vaccination confidence, either for the whole region or in specific populations within a country. For example, in a national survey in New Zealand by Massey University in July, 2020, despite 75% of participants overall agreeing to receive a COVID-19 vaccine, the willingness to receive the vaccine was lower among the Māori population and other socioeconomically disadvantaged groups than the general population.

The safety and potential side-effects of COVID-19 vaccines are often cited as the main reasons for not wanting to be vaccinated. Considering that vaccine confidence is low in some countries and populations of the Western Pacific region, a rush toward implementing mass COVID-19 vaccination programmes and the absence of long-term safety data could exacerbate the existing concerns. The resultant low vaccination rates in some populations might further widen the immunity gaps, creating pockets of unprotected communities and subsequently causing a resurgence of the disease. This story is not new in the region. According to the vaccine confidence study published in *The Lancet* in Sept 26, 2020, Japan and the Philippines ranked among the top ten countries globally with the lowest vaccine confidence for vaccine safety, importance, and effectiveness between 2015 and 2020.

Misinformation often spreads faster than robust scientific evidence. In the vast majority of cases, the reasons behind vaccine hesitancy in the region are not backed up by solid scientific data. The poorer reception towards COVID-19 vaccines in Japan and among minority groups in New Zealand suggests that more work needs to be done to better prepare the Western Pacific region for the imminent arrival of COVID-19 vaccines. In this new phase of controlling the pandemic, the transparent and robust evidence for vaccine efficacy and safety is core to winning the public's trust on vaccines, along with responsive communication strategies to deliver the correct messages, to build vaccine confidence, and to achieve the long-term goal of herd immunity through vaccination.

Vaccine hesitancy is a complex issue that requires concerted efforts from the government, scientific community, and civil society to minimise its ramifications on vaccine coverage. In this issue of *The Lancet Regional Health – Western Pacific*, Adam Jenney and colleagues report that the introduction of a rotavirus vaccine to Fiji's national vaccine programme in 2012 has reduced the morbidity and mortality from rotavirus and all-cause diarrhoea in individuals aged between 2 months and 54 years, providing solid evidence supporting the efficacy of rotavirus vaccine in Fiji. As echoed by Naor Bar-Zeev and colleagues in the linked commentary, such solid scientific evidence for vaccines is encouraging and is very welcomed in Pacific Island countries, to boost vaccine confidence and to contribute to policy making in Western Pacific countries.

*The Lancet Regional Health – Western Pacific*

